# Identification and genetic characterization by high-throughput SNP analysis of intervarietal substitution lines of rapeseed (*Brassica napus* L.) with enhanced embryogenic potential

**DOI:** 10.1007/s00122-015-2455-7

**Published:** 2015-01-28

**Authors:** Wolfgang Ecke, Anthimos Kampouridis, Katharina Ziese-Kubon, Ann-Catrin Hirsch

**Affiliations:** Department of Crop Sciences, Georg-August-Universität Göttingen, Von-Siebold-Str. 8, 37075 Göttingen, Germany

## Abstract

****Key message**:**

**Seven intervarietal substitution lines were identified with embryogenic potentials up to 40.4 times that of the recurrent parent, providing an ideal material for further in depth studies of this trait.**

**Abstract:**

To identify genomic regions that carry genetic factors controlling embryogenic potential of isolated microspores of rapeseed, marker segregations were analysed in a segregating population of haploid microspore-derived embryos and a BC_1_ population from a cross between ‘Express 617’ and ‘RS239’. After map construction 15 intervarietal substitution lines from the same cross with ‘Express 617’ as recurrent parent were selected with donor segments covering five genomic regions that had shown skewed segregations in the population of microspore-derived embryos but not in the BC_1_ population. By comparing the embryogenic potential of microspores of the 15 substitution lines and ‘Express 617’, seven lines were identified with significantly enhanced embryogenic potential ranging from 4.1 to 40.4 times that of ‘Express 617’. To improve the genetic characterization of the selected lines, they were subjected to a high-throughput SNP analysis using the Illumina Infinium 60K chip for rapeseed. Based on 7,960 mapped SNP markers, one to eight donor segments per line, which cover 0.64–6.79 % of the 2,126.1 cM of the SNP map, were found. The SNP analysis also gave evidence that homoeologous exchanges had occurred during the development of the substitution line population, increasing the genetic diversity within this population. By comparing donor segments between lines with significantly enhanced embryogenic potential and non-significant lines, 12 genomic regions were identified that may contain genetic factors controlling embryogenic potential in rapeseed. These regions range in size from 0 (represented by just one marker) to 26.8 cM and cover together just 5.42 % of the SNP map.

**Electronic supplementary material:**

The online version of this article (doi:10.1007/s00122-015-2455-7) contains supplementary material, which is available to authorized users.

## Introduction

In many crop species it is possible today to develop entirely homozygous plants in one generation, which are called doubled haploids (DHs). A commonly used approach to achieve this is androgenesis, in which a sporophytic development is induced in isolated microspores, usually by a mild heat stress. The induced microspores first develop into embryo-like structures, called “microspore derived embryos” (MDEs) that can be further grown into fully developed plants. On treatment with a mitosis inhibitor, usually colchicine, a doubling of the haploid chromosome complement of the original microspore can be achieved, leading to diploid plants (Chen et al. 1994; Möllers et al. 1994) that can be propagated by selfing as true lines (DH lines).

Androgenesis and DH lines are of great importance in research as well as practical breeding in rapeseed and related *Brassica* crops. Many rapeseed varieties currently grown are hybrids of DH lines. Also, microspores and MDEs are used for mutagenesis and in vitro selection (Albrecht et al. 1995; Barro et al. 2001; Ferrie et al. 2008; McClinchey and Kott 2008) and protocols have been developed for the genetic transformation of microspores (Guerche et al. 1987; Fukuoka et al. 1998; Nehlin et al. 2000; Dormann et al. 2001). Using microspores large numbers can be processed in the space of a petri dish and genetic changes can be fixed in one step. Furthermore, for genetic mapping in rapeseed usually F_1_ derived segregating DH populations were used from the beginning (Ferreira et al. 1994; Sharpe et al. 1995; Uzunova et al. 1995; Delourme et al. 2006; Qiu et al. 2006; Radoev et al. 2008). In addition, due to the ease with which large numbers of microspores can be isolated from rapeseed and the highly efficient induction of embryogenesis in some genotypes, rapeseed has become a model system for the analysis of early steps in microspore embryogenesis and embryo maturation, especially since there is no working protocol for androgenesis in *Arabidopsis thaliana* (Malik et al. 2007).

The primary limiting step in the production of MDEs and DH lines is the embryogenic potential of the isolated microspores, i.e., their tendency to develop into embryos after induction. The rate of embryo formation is strongly dependent on culture conditions, but even with an optimised protocol tremendous differences are observed between different genotypes that clearly indicate the involvement of genetic factors (Ferrie et al. 1995; Barro and Martín 1999; Kuginuki et al. 1999; Ferrie and Keller 2007).

In an early study on the genetics of embryogenic potential Foisset et al. (1993) analysed the segregation of ten isoenzyme markers in MDEs of five rapeseed crosses. In two crosses five of the markers showed significant deviations from the expected segregation ratio that were not observed in corresponding F_2_ populations. Under the hypothesis that the segregation of alleles conferring different levels of embryogenic potential leads to a selection of favourable alleles during microspore embryogenesis the skewed marker segregations would indicate that these markers are linked to genes controlling embryogenic potential in rapeseed. In a similar study Tanhuanpää et al. (1994) found skewed segregations for five out of 25 RAPD markers in a segregating microspore-derived population that were also not observed in a corresponding F_2_ population. By comparing segregation patterns of markers in two segregating DH populations and an F_2_ population from a cross between two rapeseed genotypes with strongly different embryogenic potential, Cloutier et al. (1995) identified two regions on two linkage groups showing an excess of marker alleles of the better parent, indicating that these regions could carry genes controlling embryogenic potential.

Skewed marker segregations have actually been observed in many mapping studies using DH populations in rapeseed (reviewed in Ferrie and Möllers 2011). Usually, these markers cluster on the map, defining one or more genomic regions with skewed segregations, each favouring a particular parental allele. Across different studies, regions with skewed segregations have been found on all linkage groups of the rapeseed genome. In many of these studies the hypothesis was posited that these regions carry genes controlling embryogenic potential. While it is plausible that the segregation of alleles conferring different levels of embryogenic potential will lead to skewed segregations, it is not the only factor that can cause such segregation patterns. During the development of DH populations there are additional limiting steps, like diploidization or the embryo to plant conversion (Möllers and Iqbal 2009) where selection at loci controlling these steps can cause skewed segregations. In addition, skewed segregations can also occur just by chance due to sampling effects. Only based on marker analyses it is not possible to distinguish between these possibilities. To prove that skewed segregations are due to selection at loci controlling embryogenic potential an independent verification is necessary but in the studies cited above such a verification was not carried out. Only Zhang et al. (2003), who had identified RAPD markers with skewed segregations in a microspore-derived population of rapeseed, verified the linkage of these markers to genes controlling embryogenic potential by determining the embryogenic potential of all plants in a corresponding F_2_ population. They found that three RAPD markers were actually associated with this trait. In the same way the authors identified seven RAPD markers in *B. rapa* that were linked to genes controlling embryogenic potential in that species. Unfortunately, although the authors did map the RAPD markers, the linkage groups were not assigned to the known linkage groups of rapeseed. Accordingly, the results of Zhang et al. are difficult to relate to results from other mapping studies.

The lack of confirmation that genomic regions showing skewed segregations in microspore-derived populations and regular segregations in F_2_ populations actually carry genes involved in the control of embryogenic potential is probably due to the difficulty to determine the embryogenic potential of all genotypes of a mapping population large enough to map the genes or QTL underlying this trait. This problem could be circumvented using intervarietal substitution lines (ISLs, Howell et al. 1996) for verification. ISLs are developed by marker-assisted selection during a recurrent backcrossing program, starting with a cross between a donor and a recurrent parent. Each ISL carries one or a few segments of the donor genome in the common genetic background of the recurrent parent. Across all lines, large parts of the donor genome are represented. By selecting ISLs with donor segments covering regions that had shown skewed segregations in a corresponding microspore-derived segregating population the presence of genes controlling embryogenic potential in these regions could be verified by determining the embryogenic potential of the selected lines and comparing it with the potential of the recurrent parent. This way, the embryogenic potential would have to be determined for only a small number of genotypes. The objective of this study was to use this approach to find ISLs with embryogenic potentials significantly different from the embryogenic potential of the recurrent parent and to identify genomic regions responsible for these differences.

## Materials and methods

### Plant materials, genetic maps

Skewed marker segregations were analysed in a segregating population of 191 haploid MDEs from one F_1_ plant of a cross between ‘Express 617’, an inbred line of the winter rapeseed variety ‘Express’, and ‘RS239’, a resynthesized rapeseed line. The F_1_ plant had been clonally propagated in vitro to provide multiple donor plants for microspores. The same F_1_ plant was also backcrossed as male parent to ‘Express 617’ to develop a BC_1_ population of 185 plants.

For verification of the effects of specific genomic regions on the embryogenic potential of microspores a population of intervarietal substitution lines was available. The ISL population consists of 323 doubled haploid lines derived from microspores of BC_4_ plants from a cross between the same parents as the MDE population. During the development of the ISL population ‘Express 617’ had been the recurrent parent. To identify donor segments in the ISL population, the population had been analysed with 484 AFLP markers from 5 primer sets (20 primer combinations), which had been mapped in a corresponding segregating DH population of the same cross. In addition to the AFLP markers, 240 SSR markers had also been mapped in the DH population, which allowed identifying the linkage groups of the resulting map (DH map) and name them according to the consensus nomenclature proposed by the Steering Committee of the Multinational Brassica Genome Project (http://www.brassica.info/resource/maps/lg-assignments.php). The SSR marker data had been kindly provided by KWS SAAT AG, Einbeck, Germany and the DH population by Norddeutsche Pflanzenzucht Hans-Georg Lembke KG, Hohenlieth, Germany. According to the AFLP analysis the ISLs carry between one and nine donor segments, with an average of three, which in total cover a minimum of 951 cM and a maximum of 1,587 cM of the 2,003 cM of the DH map (Nurhasanah 2010). The minimum length of a donor segment was calculated as the distance between the first and last marker of the segment showing donor alleles, the maximum length as the distance between the last marker before the segment that still showed the allele of the recurrent parent and the first marker after the segment that showed a recurrent parent allele. If the beginning or end of a segment coincided with one end of a linkage group, minimum and maximum extent were calculated from that same point. Donor segments represented by only one marker were arbitrarily assigned a minimal length of 0 cM.

For the SNP analysis, an SNP map developed in a segregating DH population from a cross between ‘SGDH14’ and ‘Express 617’ was kindly provided by Christian Möllers and Nina Behnke. ‘SGDH14’ is a line from a DH population of a cross between ‘Gaoyou’, a Chinese semi-winter rapeseed variety and ‘Sollux’, a German winter rapeseed variety (Zhao et al. 2005). The SGDH14xE map was constructed using JoinMap after a high-throughput SNP analysis carried out as service by TraitGenetics GmbH, Gatersleben, Germany. The map is comprised of 15,474 SNP markers distributed across 19 linkage groups and has a total length of 2,126 cM.

### Microspore isolation and cultivation

Microspore donor plants were grown in the greenhouse. When the first flower buds appeared, the plants were transferred to an environmentally controlled growth chamber and further cultivated under a photoperiod of 16 h with day/night temperatures of 12° C/6° C (Iqbal et al. 1994). Flower buds 2–3 mm in length were harvested, surface sterilized under moderate agitation for 5–20 min in 1 % Ca hypochlorite laced with some Tween 20, and finally washed three times with cold sterile water. Sixteen flower buds were crushed with a pistil on a sieve placed in a 9 cm petri dish filled with 5 ml NLN medium [0.039 % (w/v) NLN medium basal salt mixture, 0.104 % (w/v) NLN medium vitamin mixture (Duchefa Biochemie B.V., Haarlem, The Netherlands), 0.05 % (w/v) Ca(NO_3_)_2_·4H_2_O (Sigma-Aldrich Laborchemikalien GmbH, Seelze, Germany), 13 % (w/v) sucrose]. After rinsing the pistil and sieve with 7 ml NLN medium, the medium now containing the microspores was transferred into a glass centrifuge tube and centrifuged for 5 min at 1,000 rpm. The microspore pellet was resuspended in 12 ml NLN medium and centrifuged again. After repeating the washing step once, the final pellet was resuspended in 5 ml NLN medium and transferred to a 9 cm petri dish already containing 7 ml NLN medium. The petri dish was sealed with two layers of Parafilm and incubated for 7 days at 32 °C in the dark followed by incubation for 7 days at 28 °C, also in the dark. Afterwards the microspores were cultivated under slight agitation in light with a photoperiod of 12 h at a constant temperature of 22 °C for 3 weeks after which the number of MDEs that had developed in a culture was counted.

After microspore isolation the microspore density in the microspore culture was determined using a Fuchs-Rosenthal haemocytometer. Two values were calculated: the total density of microspores in the culture and the density of microspores at the optimal stage for induction of embryogenesis, that is at the late uninucleate or early binucleate stage (Pechan and Keller 1988). The embryogenic potential was then expressed as the number of MDEs derived from 10^6^ microspores at the optimal stage.

During the development of the segregating MDE population ploidy levels of MDEs were determined by flow cytometry from 0.5 cm^2^ samples of single cotyledons. For flow cytometry a Partec Ploidy Analyser PA-I (Partec GmbH, Görlitz, Germany) was used and ploidy levels were evaluated according to the protocol provided by the manufacturer. Haploid MDEs were then frozen in liquid nitrogen and stored at −20 °C until DNA preparation.

### DNA isolation, marker analysis and map construction

DNA was prepared from individual MDEs or 0.1 g of leaf material of 3-week-old greenhouse-grown plants using Nucleon PhytoPure extraction kits (RPN8510, GE Healthcare Life Sciences, Freiburg, Germany), following the manufacturer’s instructions. AFLP analyses were carried out following the multiplexing protocol described in Ecke et al. (2010).

The fit of marker segregations to 1:1 and 3:1 segregation ratios were tested by *χ* ^2^ tests. Significant deviations were declared at *P* = 0.05. The *χ* ^2^ values from the test for 1:1 segregation were also used as a measure for the degree to which marker segregations deviate from the expected 1:1 ratio.

Genetic maps in the MDE and BC_1_ populations were constructed using MAPMAKER/EXP 3.0 b and a Perl script that automates the mapping process and distributes time critical processes to multiple copies of MAPMAKER to speed up the analysis. For map construction a mapping approach was used in which only a subset of markers, which can be ordered with a log-likelihood support of at least 3.0 and a maximal distance of 35 cM, are actually mapped to form a high fidelity (HF) map. All other markers are only placed at their most likely position alongside the HF map to form the full map. During the development of the HF map double crossovers (DC) were used as proxy for marker quality since most scoring errors would appear as DC in a DH or BC_1_ population. Markers whose number of DC exceeded the expected number by more than 3 were excluded from the HF map. The expected number was roughly estimated by multiplying the product of the recombination frequencies between a marker and its two flanking markers with the number of informative genotypes. In addition, markers with strongly skewed segregations, that is markers with segregation ratios not significantly different from a 3:1 ratio or beyond, were initially excluded from the mapping. These markers were mapped or placed in a second pass only when they (1) showed significant linkage to markers of only one linkage group in a two-point analysis and (2) were not farther apart from the next marker than 20 cM. This two-pass mapping approach was mainly designed to exclude bands from the mapping that represent a superposition of the segregation of two marker loci. AFLP primer pairs amplify many loci simultaneously and occasionally two loci may produce similar-sized products that cannot be distinguished in the analysis. In addition, in rapeseed it is possible that sometimes two homoeologous loci produce the same amplification product. The resulting band will segregate in a 3:1 fashion. The corresponding loci cannot be properly mapped but the band will still show linkage to the two sets of loci that are linked to the underlying loci, which will cause problems in the mapping process.

After map construction the consistency of the linkage groups was verified using LOD tables with LOD scores derived from Chi-square tests for the independence of two segregations, conditional on their marginal frequencies (Mather 1957; Lombard and Delourme 2001). If necessary, the automatically generated maps were modified working interactively with MAPMAKER.

High-throughput SNP analyses using the Illumina Infinium 60K chip for rapeseed were provided as service by TraitGenetics GmbH.

### Statistical analysis

Statistical analyses were carried out with the program Statistica version 10 (StatSoft. Inc., Tulsa, OK, USA). Dunnett’s test was applied to test for differences in the embryogenic potential between ISLs and the recurrent parent ‘Express 617’ at *P* = 0.05. The Kolmogorov–Smirnov test with the D statistic interpreted according to Lilliefors (1967) was used to test for normal distribution within groups. To test for variance homogeneity Levene’s test was applied. If normal distribution or variance homogeneity was not met the data were log_10_ transformed after increasing all values by 1 to accommodate zero values [log_10_(*x* + 1)].

## Results

### Genetic mapping in MDE and BC_1_ population

Using the same 5 primer sets that had been used to construct the DH map and characterise the ISL population 535 AFLP markers could be scored in the MDE population. Using these markers, a map comprising 481 markers was constructed (Table S1). The MDE map consists of 20 linkage groups that could be assigned to the 19 chromosomes of rapeseed based on shared markers with the DH map and a map from a cross between ‘Express 617’ and ‘R53’ (Ecke et al. 2010). A2 was present in two disjointed parts and C4 only as a short fragment of four markers. In addition, three unjoined marker triplets and one pair were observed. The full map had a length of 1,943.2 cM (Table 1). In the BC_1_ population 271 AFLP markers were scored and a map of 221 markers could be developed. In this map C4 was missing altogether and C7 was present as three disjointed parts. Full map length here was 1,327.4 cM (Table 1).

**Table 1:** Linkage groups, number of markers and length of linkage groups of the high fidelity (HF) and full maps in the MDE and BC_1_ populations

LG	MDE population				BC_1_ population			
	HF map		Full map		HF map		Full map	
	No. markers	Length (cM)	No. markers	Length (cM)	No. markers	Length (cM)	No. markers	Length (cM)
A01	16	91.7	20	91.7	11	76.8	16	76.8
A02a^a^	16	104.2	23	104.2	8	55.5	12	55.5
A02b	5	22	11	22				
A03	41	125	46	125	14	134.5	21	152.5
A04	14	108.4	16	108.4	3	9.5	5	28.3
A05	24	60.7	29	60.7	11	65	17	65
A06	24	112.8	25	112.8	7	39.6	9	40.2
A07	16	88.9	20	88.9	11	88.7	11	88.7
A08	17	62.4	19	62.4	8	61.3	9	62.2
A09	26	114.8	28	114.8	10	86.5	13	86.5
A10	14	74.2	16	74.2	8	79.7	9	79.7
C01	17	108.9	23	134.8	10	60.4	11	60.4
C02	16	85.8	23	85.8	6	72.9	7	72.9
C03	28	149.1	30	149.1	12	122.1	17	122.1
C04	4	17.1	4	17.1	–	–	–	–
C05	26	83.5	32	83.5	12	58.2	18	58.2
C06	20	94.6	22	94.7	9	81.2	11	90.5
C07a^b^	23	126	25	126	5	16.1	7	18.4
C07b					2	2.2	2	2.2
C07c					2	3.4	2	3.4
C08	20	114.2	20	114.2	8	70	9	70
C09	32	127	38	127	13	91.1	13	91.1
Naa	2	12.3	3	14.5				
Nbb	2	1.6	3	20.4	2	2.8	2	2.8
Ncc	2	8.3	2	8.3				
Ndd	3	2.7	3	2.7				
Sum	408	1,896.2	481	1,943.2	172	1,277.5	221	1,327.4

^a^A2 is present in two disjointed parts in the MDE map but is continuous in the BC_1_ map

^b^C07 is present in three disjointed parts in the BC_1_ map but is continuous in the MDE map

### Analysis of skewed segregations

Of the 481 markers on the MDE map, 230 (48 %) showed a significant deviation from the expected 1:1 segregation ratio. Markers with skewed segregations were found on all linkage groups with the exception of A3, C4 and the unjoined pair (Table S1). Eight markers were found on the unjoined triplets and were omitted from further analysis since the triplets could not be aligned to the DH map. Most of the remaining 222 markers clustered in 22 regions with 2–28 markers, including a few markers with regular segregations, covering from 0.5 to 97.7 cM. In total these regions represent 654.2 cM (33.7 %) of the map. In addition, 12 single markers with skewed segregations were observed, indicating small regions with disturbed segregations, increasing the total number of these regions to 34 (Table 2). Fifteen regions showed excess of ‘Express 617’ alleles and 19 an overabundance of ‘RS239’ alleles with the peak *χ* ^2^ values ranging from 3.86 to 51.86.

**Table 2:** Regions with skewed segregations on the MDE map and comparison with the corresponding regions on the BC_1_ map

No.	LG	MDE map						BC_1_ map					
		Start (cM)	End (cM)	Length (cM)	tm/sm	Favoured allele	Peak *χ* ^2^	Start (cM)	End (cM)	Length (cM)	tm/sm	Peak *χ* ^2^	Favoured allele
1	A01	0.0	0.5	0.5	2/2	R	12.13	41.0	41.5	0.5	2/0	0.56	–
2	A01	50.7	67.8	17.1	6/5	R	5.14	5.7	25.9	20.2	6/0	1.26	–
3	A02a	0.0	69.1	69.1	14/12	R	32.71	0.0	24.9	24.9	3/0	1.42	–
4	A02a	104.2	104.2	0.0	1/1	R	8.99	–	–	–	–	–	–
5	A02b	0.0	22.0	22.0	11/11	R	36.45	46.1	55.5	9.4	9/0	2.49	–
6	A04	22.7	108.4	85.7	15/15	R	28.81	−18.0	10.8	28.8	5/3	28.16	R
7	A05	17.8	60.7	42.9	28/28	E	32.40	13.6	65.0	51.4	15/2	6.92	R
8	A06	25.5	42.2	16.7	4/4	E	9.38	–	–	–	–	–	–
9	A06	65.9	65.9	0.0	1/1	E	5.51	0.0	0.0	0.0	1/0	0.14	–
10	A06	106.3	106.3	0.0	1/1	E	3.86	39.6	39.6	0.0	1/0	0.57	–
11	A07	10.0	54.7	44.7	12/10	E	20.45	33.1	85.0	51.9	7/0	2.22	–
12	A07	73.4	73.4	0.0	1/1	E	3.98	–	–	–	–	–	–
13	A08	8.9	8.9	0.0	1/1	E	3.94	53.0	53.0	0.0	1/0	0.36	–
14	A08	27.6	62.4	34.8	6/6	E	26.34	0.0	29.5	29.5	2/0	1.14	–
15	A09	0.0	0.0	0.0	1/1	R	5.82	0.0	0.0	0.0	1/0	0.57	–
16	A09	24.2	40.7	16.5	2/2	E	10.71	6.4	22.8	16.4	2/0	1.24	–
17	A09	87.8	114.8	27.0	18/18	R	23.10	62.8	86.5	23.7	7/6	8.5	E
18	A10	0.0	27.9	27.9	10/9	R	8.50	0.0	24.5	24.5	5/0	0.14	–
19	C01	−25.9	−25.9	0.0	1/1	R	16.57	–	–	–	–	–	–
20	C01	48.1	61.3	13.2	6/6	R	9.28	–	–	–	–	–	–
21	C01	95.4	95.4	0.0	1/1	R	5.14	–	–	–	–	–	–
22	C02	7.4	46.6	39.2	15/15	E	51.86	27.1	27.1	0.0	1/0	0.09	–
23	C02	62.0	62.0	0.0	1/1	E	4.36	–	–	–	–	–	–
24	C02	85.8	85.8	0.0	1/1	R	5.06	72.9	72.9	0.0	1/0	0.46	–
25	C03	0.0	14.0	14.0	2/2	R	11.07	14.5	21.7	7.2	2/0	0.79	–
26	C03	77.7	125.5	47.8	14/12	R	10.33	80.1	114.8	34.7	7/0	1.63	–
27	C05	38.7	39.8	1.1	8/7	E	4.74	19.4	20.6	1.2	5/0	0.92	–
28	C06	0.0	94.7	94.7	22/22	E	48.02	0.0	90.5	90.5	11/1	4.65	E
29	C07	7.5	23.0	15.5	10/9	R	8.51	7.0	10.6	3.6	3/2	5.63	E
30	C07	123.7	123.7	0.0	1/1	R	5.00	0.0	0.0	0.0	1/0	1.41	–
31	C08	114.2	114.2	0.0	1/1	R	30.14	–	–	–	–	–	–
32	C09	0.0	13.9	13.9	6/6	R	9.78	91.1	91.1	0.0	1/0	0.79	–
33	C09	64.5	70.2	5.7	8/6	E	7.32	27.6	34.2	6.6	5/0	0.58	–
34	C09	98.1	102.3	4.2	3/3	E	5.76	0.0	0.0	0.0	1/0	0.2	–
Sum				654.2	234/222					425.0	105/14		

*tm* total number of markers in the region, *sm* number of markers with significantly skewed segregation (*P* = 0.05) in the region, *R* ‘RS239’ allele, *E* ‘Express 617’ allele

With only 12 % (26 markers) the percentage of markers with skewed segregations was much lower on the BC_1_ map. Of the 34 regions with skewed segregations on the MDE map eight were not covered in the BC_1_ map and for the remaining 26 regions 21 of the corresponding regions on the BC_1_ map carried only markers with regular segregations. Only five of the corresponding regions showed markers with skewed segregations in the BC_1_ population, too, but in three cases the favoured allele was different (Table 2; Table S1). In addition, on average the fraction of markers with skewed segregations in these regions and the peak *χ* ^2^ values were lower in the BC_1_ population than in the MDE population.

### Selection of ISL with donor segments covering selected regions with skewed segregations

**Fig. 1:**
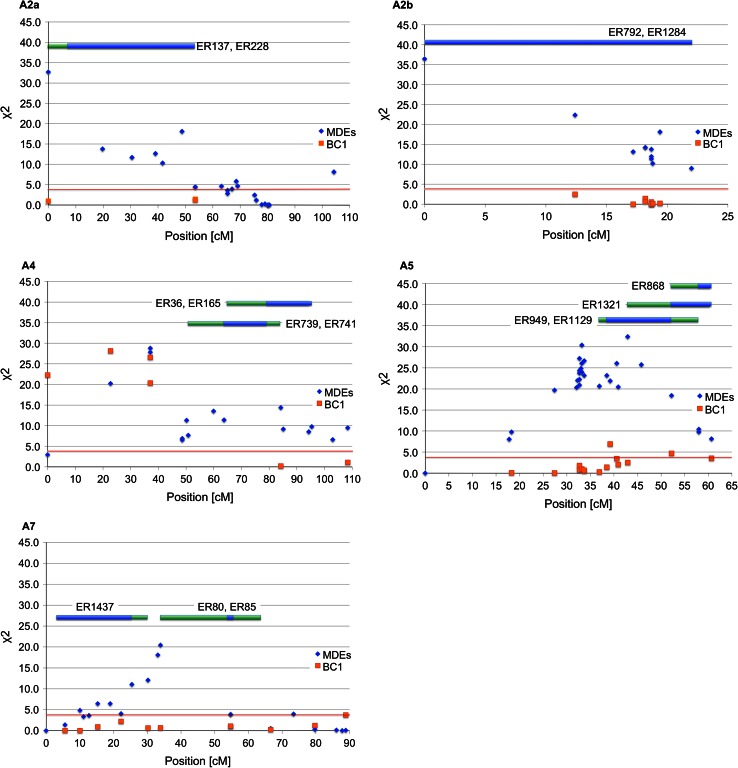
Distribution of marker segregation patterns in the MDE and BC_1_ populations and donor segments of selected ISLs on linkage groups A2a, A2b, A4, A5 and A7 of the MDE map. Values from a *χ* ^2^ test for 1:1 segregation are displayed as a measure for the degree to which marker segregations deviate from the expected segregation ratio. The *horizontal red line* indicates the significance threshold at *P* = 0.05. Each *blue* and *red* mark represents a marker on the MDE and BC_1_ map, respectively. Positions refer to the MDE map. The *blue* and *green* bars represent minimal and maximal extents, respectively, of the donor segments of selected ISL on the five linkage groups. The donor segments of ISLs ER80 and ER85 on A7 are represented by only one marker and have a nominal minimal extent of 0 cM. In the figure the minimal extent has been drawn larger for the sake of visibility

Based on peak *χ* ^2^ values, the distribution of skewed segregations across the regions, and the availability of ISLs with donor segments covering the regions, five regions on A2a, A2b, A4, A5 and A7 (nos. 3, 5, 6, 7 and 11 in Table 2) with skewed segregations showing high peak *χ* ^2^ values were chosen for further analysis (Fig. 1). For three of these regions, on A2a, A2b and A7, the corresponding regions on the BC_1_ map did not show any skewed segregations. For the region on A5, two markers in the corresponding region of the BC_1_ map showed skewed segregations, but at these loci the ‘RS239’ allele was favoured, not the ‘Express 617’ allele as in the MDE population, indicating a difference in the cause for the disturbed segregations in the two populations. With 85.7 cM the region with skewed segregations on A4 was rather large. In the upper part of this region four markers also showed strongly skewed segregations in the BC_1_ population, favouring the same allele as in the MDE population. On the other hand, the two markers in the lower part of the regions that had also been mapped in the BC_1_ population showed regular segregations, although in the MDE population markers in this part still gave *χ* ^2^ values as high as 14.38 (Fig. 1). Taking this distribution of segregation patterns into account, only ISLs with donor segments covering the lower part of the region on A4 were selected.

In total, 15 ISL with fitting donor segments were selected. On A2b the donor segments of the two selected ISLs cover the whole linkage group and on A2a between 64 and 77 % (minimal to maximal extent of the donor segments) of the region with skewed segregations. On A4 and A5 the donor segments of the four selected ISLs cover about half of the region with skewed segregations although on A4, where only the lower half of the region was of interest, the coverage of this part of the region was much higher. On A7 there was a strong discrepancy between the coverage seen by the minimal and maximal extent of the donor segments of the three selected ISLs. Taking into account just the minimal extent only 41 % of the region with skewed segregations was covered, but with the maximal extent of the donor segments the coverage would increase to 92 %. The minimal and maximal extents of the donor segments covering the selected regions with skewed segregations are shown in Fig. 1 with respect to the MDE map.

### Determination of the embryogenic potential of the selected ISL

To determine the embryogenic potential of isolated microspores of the selected ISLs and compare it to the embryogenic potential of the recurrent parent ‘Express 617’, the embryogenic potential was evaluated in 10–23 independent microspore cultures per genotype (Table 3). With a mean value of just 11.9 ‘Express 617’ showed a rather low embryogenic potential. Among the ISL the mean values ranged from 2.8 to 482.3, which represent a range from 0.2 to 40.4 times the embryogenic potential of the recurrent parent of the ISLs. Based on log_10_ transformed data the seven ISLs ER36, ER85, ER137, ER165, ER228, ER1129, and ER1321 showed significantly higher embryogenic potentials than ‘Express 617’, with ER85 showing the highest potential, followed by ER228. ISLs with significantly lower embryogenic potential were not observed.

**Table 3:** Comparison of the embryogenic potential of 15 selected ISLs to the embryogenic potential of ‘Express 617’ ISL

ISL	*n* ^a^	Mean^b^	Ratio	Minimum	Maximum	Stdev^c^
ER36	12	48.8**	4.1	1.6	162.0	53.77
ER80	12	34.8^n.s.^	2.9	0.0	88.7	30.43
ER85	13	482.3***	40.4	161.9	1,048.4	265.09
ER137	15	138.4***	11.6	0.0	602.6	198.45
ER165	18	50.2*	4.2	0.0	173.4	56.32
ER228	10	345.8***	28.9	47.2	834.2	230.73
ER739	19	2.8^n.s.^	0.2	0.0	12.9	3.94
ER741	17	9.5^n.s.^	0.8	0.0	43.8	14.72
ER792	14	15.7^n.s.^	1.3	0.0	69.8	17.81
ER868	23	5.7^n.s.^	0.5	0.0	32.0	9.83
ER949	14	26.1^n.s.^	2.2	0.0	74.2	25.95
ER1129	12	110.6***	9.3	0.0	277.7	81.10
ER1284	19	9.4^n.s.^	0.8	0.0	76.6	17.37
ER1321	13	93.7***	7.8	5.2	506.7	135.99
ER1437	18	10.4^n.s.^	0.9	0.0	52.8	14.00
Express 617	21	11.9	1.0	0.0	55.5	16.19

^a^Number of microspore cultures

^b^Embryogenic potential is expressed as the number of embryos derived from 10^6^ cultivated microspores at the optimal developmental stage, *,**,***: Significantly different from ‘Express 617’ at *P* = 0.05, 0.02 and 0.01, respectively, n.s.: not significant

^c^Standard deviation

### High-throughput SNP analysis

To improve the genetic characterization of the selected ISLs, the lines were subjected to a high-throughput SNP analysis. In total, 52,157 SNP markers were analysed in the 15 ISLs, the two parents and the F_1_. Of these 6,418 were removed from further analysis because they gave only failed or ambiguous scorings in all ISL or the genotypes of the parents could not be determined. Of the remaining 45,739 markers, 21,373 (46.73 %) proved to be polymorphic between ‘Express 617’ and ‘RS239’ with 7,960 of the polymorphic markers having also been mapped on the SGDH14xE map. The mapped markers cover 2,109.7 cM or 99.2 % of this map. A genotypic characterization of the 15 ISL based on the 7,960 mapped SNP markers is shown in Table S2 and Figure S1 provides an overview of donor segments and marker scorings across the linkage groups of the SGDH14xE map.

In total, 66 distinct, sometimes overlapping segments were detected by the SNP analysis in the 15 ISLs, distributed across 16 linkage groups (Table S3). In four cases a small segment following a larger segment was indicated by the sequence position of the markers as provided in the marker names to be part of the larger segment. In the final compilation of donor segments (Table 4) each of these segment pairs was considered as one continuous segment. Not all segments showed the expected structure of donor segments where a continuous region of the genome shows donor genotypes. Eight segments on linkage groups C1, C2, C6 and C8 carried blocks of “failed scores”. A manual inspection of these marker scores showed in nearly all cases a very low signal intensity of the marker assay, indicating the actual absence of the marker from the affected genotypes (Jana Lemm, TraitGenetics, personal communication). In addition, at the beginning of A1 a cluster of eight small segments was observed over a range of 26.8 cM in ER1321. The first of these segments again shows failed scores. Due to the close spacing of these segments they were considered as one large segment in the further analysis (Table 4).
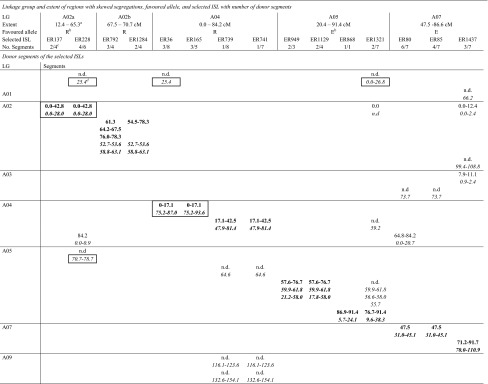
 
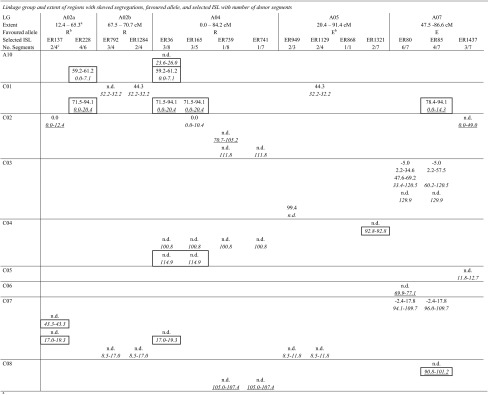

**Table 4:**
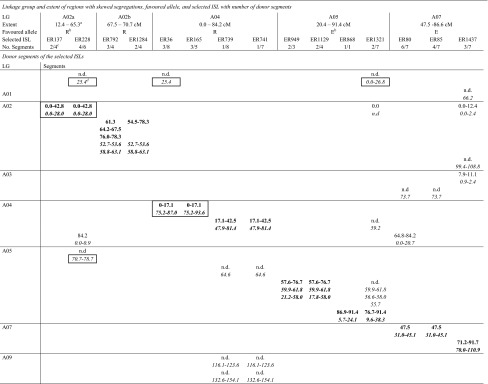

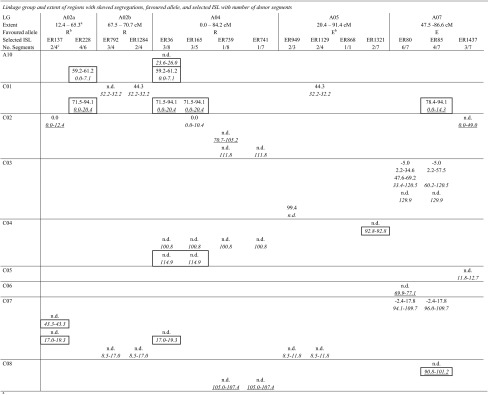
Regions with skewed segregations in the MDE population and donor segments of intervarietal substitution lines selected for coverage of these regions

^a^Extent of genomic regions showing skewed segregations in the MDE population. Positions are in cM. The positions refer the DH map and were derived from a map alignment with the MDE map

^b^R, E: excess of ‘RS239’ or ‘Express 617’ alleles

^c^Number of donor segments according to DH map/SGDH14xE map

^d^Minimal extent of donor segments with positions in cM. Positions in regular typeface indicate donor segments detected by the original AFLP analysis and refer to the DH map. Positions in italics indicate donor segments detected by SNPs mapped on the SGDH14xE map. Corresponding segments are listed above one another with n.d. indicating that a segment was not detected in the respective analysis. Segments in bold were selected to cover the regions with skewed segregations. Segments underlined represent segments with blocks of markers showing failed scores. Boxed segments cover or overlap the genomic regions that may be involved in the enhanced embryogenic potential of the significant lines

The number of segments detected by the SNP analysis in the different ISLs ranged from one to eight (Table 4). The segments in individual ISLs cover in their minimal extent between 13.7 and 144.3 cM of the SGDH14xE map, corresponding to 0.64–6.79 % of the total length of this map. Considering the maximal extent of the donor segments the coverage increases to 1.36–7.57 % (Table 5).

**Table 5:** Minimal and maximal genome coverage by donor segments in individual ISL according to the SNP analysis

ISL	Minimal extent		Maximal extent	
	Absolute coverage (cM)	Percentage of map (%)	Absolute coverage (cM)	Percentage of map (%)
ER36	44.0	2.07	61.2	2.88
ER80	144.3	6.79	152.6	7.18
ER85	112.4	5.29	130.7	6.15
ER137	42.7	2.01	57.9	2.72
ER165	49.2	2.31	61.2	2.88
ER228	64.4	3.03	72.5	3.41
ER739	99.4	4.68	124.5	5.86
ER741	64.9	3.05	87.7	4.12
ER792	13.7	0.64	28.9	1.36
ER868	18.4	0.87	41.2	1.94
ER949	42.0	1.98	72.9	3.43
ER1129	45.4	2.14	78.8	3.71
ER1284	13.7	0.64	28.9	1.36
ER1321	32.0	1.51	61.9	2.91
ER1437	104.1	4.90	161.0	7.57
Mean	59.4	2.79	81.5	3.83

In the AFLP analysis, between one and six donor segments had been detected in the 15 selected lines (Table 4). Because there were no shared markers between the DH and SGDH14xE map, a map alignment was not possible. Nevertheless, due to the distinct pattern of donor segments across the 15 ISLs, segments found in the AFLP analysis could be assigned unambiguously to segments identified in the SNP analysis (Table 4). There were two segments detected in the AFLP analysis, at 0.0 cM on A2 in ER1321 and at 99.4 cM on C3 in ER949, for which no corresponding segments were found in the SNP analysis. On the other hand, 29 additional donor segments were observed in the SNP analysis that had not been detected in the AFLP analysis. Most of these segments were small with minimal lengths of zero to a few cM, but one large segment of 21.5 cM on A9 was detected in lines ER739 and ER741. In addition, with the exception of the segments on C1 and one of the segments on C2, the segments with blocks of failed scores had also not been found by the AFLP markers, although some of these are rather large.

About 0.8 % of the marker genotypes in Table S2 show a failed scoring, indicating that the marker assay did not work in the respective genotype, or a heterozygote scoring, which should not occur since the ISLs are doubled haploids. These irregular marker scores are not randomly distributed. First, there are strong differences in the rate of these scorings between the genotypes, ranging from just 0.16 % in ER1284 to 2.76 % in ER1437, a 17-fold difference. Second, many of these scorings cluster in two distinct patterns. The first pattern is a horizontal clustering (with respect to Table S2 and Fig. S1), where a single marker shows failed and/or heterozygous scorings in several genotypes. Examples are the markers Bn-A01-p10693623 at 41.3 cM on A1 and Bn-A03-p20007146 at 30.3 cM on A3 (Table S2). The second pattern is a vertical clustering where several-to-many irregular scorings cluster in a specific region on a linkage group in one or a few of the ISLs. Such a cluster is, for example, found on A1 between 22.0 and 26.8 cM in ER36, ER165 and ER228. Another, much longer cluster is present in ER1129 along the whole length of A4 (Fig. S1).

### Donor segments involved in the control of embryogenic potential

**Fig. 2:**
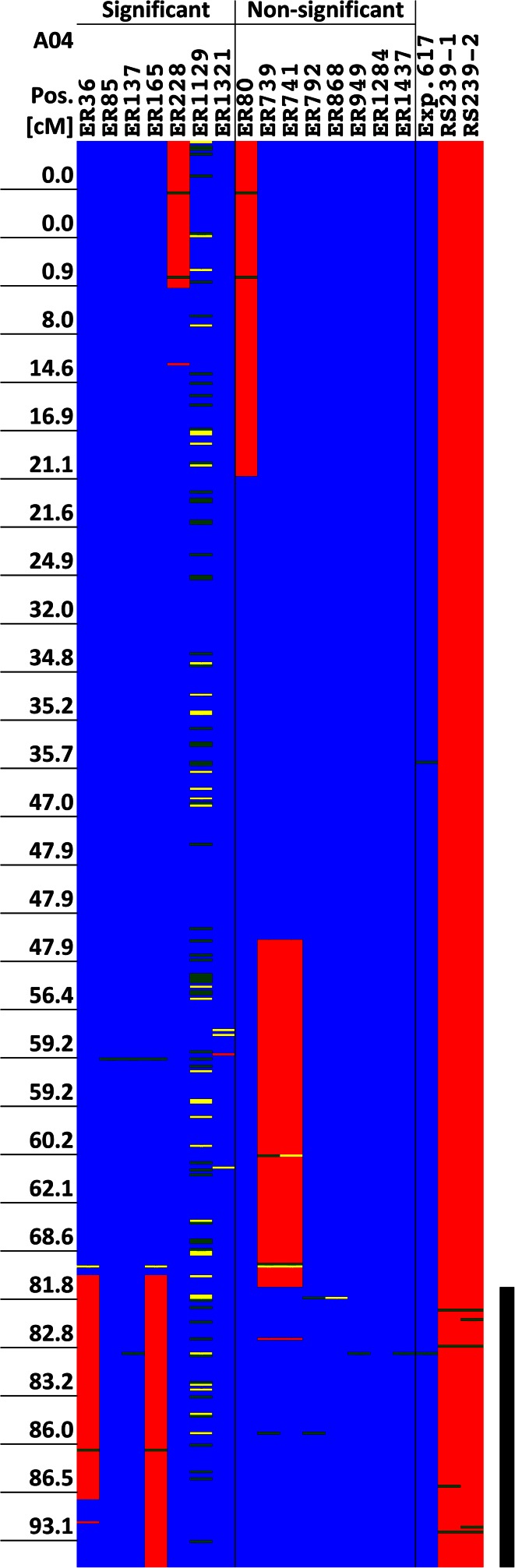
Graphical representation of donor segments and marker scorings in the selected ISLs and the parents on linkage group A4 of the SGDH14xE map. *Blue* indicates recurrent parent genotype, *red* donor genotype, *yellow* heterozygote scoring and *green* failed scoring. ‘Significant’ indicates ISLs with significantly enhanced embryogenic potential compared to ‘Express 617’, ‘Non-significant’ ISLs with embryogenic potential not significantly different from ‘Express 617’. The *black vertical bar* represents a genomic region that is only covered by donor segments in the significant lines and, therefore, may be involved in the control of embryogenic potential in rapeseed. Exp.617 is the recurrent parent ‘Express 617’, RS239-1 and RS239-2 are two replicates of the same DNA sample of ‘RS239’ in the SNP analysis. Marker positions are given for every 20th marker

By comparing the distribution and overlap of donor segments occurring in the ISLs with a significantly enhanced embryogenic potential with segments occurring in non-significant lines, 12 genomic regions on nine linkage groups could be delineated that may carry genetic factors responsible for the enhanced embryogenic potential of the significant lines. An example of this analysis is shown for the segments on linkage group A4 in Fig. 2. The 12 genomic regions range in size from 0.0 to 26.8 cM and cover a minimum of 115.2 cM (5.42 %) and a maximum of 159.8 (7.52 %) of the SGDH14xE map (Table 6). Apart from regular donor segments these regions also carry segments with blocks of failed scores on C1 and one of these segments on C8 (90.8–101.2 cM, Table 4) as well as the fragmented segment in ER1321 on A1. Individual ISLs with significantly enhanced embryogenic potential carry between 2 and 7 segments that cover or overlap these regions with the exception of ER1129, which carries none.

**Table 6:** Genomic regions that may control embryogenic potential

LG	Minimal extent			Maximal extent			No. markers	ISLs
	Start (cM)^a^	End (cM)	Length (cM)	Start (cM)	End (cM)	Length (cM)		
A01	0.0	26.8	26.8	0.0	26.8	26.8	46	ER36, ER228, ER1321
A02	2.4	28.0	25.6	2.4	28.9	26.5	7	ER137, ER228
A04	81.4	93.6	12.2	74.3	93.6	19.3	121	ER36, ER165
A05	70.7	78.7	8.0	69.8	80.1	10.3	28	ER228
A10	0.0	7.1	7.1	0.0	7.1	7.1	33	ER36, ER228
A10	23.6	26.0	2.4	23.6	26.5	2.9	19	ER36
C01	0.0	20.4	20.4	0.0	21.4	21.4	60	ER36, ER85, ER165, ER228
C04	92.8	92.8	0.0	88.5	95.1	6.6	2	ER1321
C04	114.9	114.9	0.0	107.3	115.4	8.1	1	ER36, ER165
C07	17.0	19.3	2.3	11.8	19.8	8.0	12	ER36, ER137
C07	43.3	43.3	0.0	41.3	44.7	3.4	2	ER137
C08	90.8	101.2	10.4	85.6	105.0	19.4	26	ER85
Sum			115.2			159.8		
Map coverage			5.42 %			7.52 %		

^a^Positions refer to SGDH14xE map

## Discussion

To identify genomic regions that could carry genetic factors controlling embryogenic potential in isolated microspores of rapeseed skewed marker segregations were compared between a segregating population of haploid MDEs and a corresponding BC_1_ population after constructing genetic maps in both populations. Although the same primer sets were used, with 221 markers the BC_1_ map was comprised of less than half of the 481 markers of the MDE map. This was a result of using AFLP markers for map construction, which are dominant markers. Accordingly, only markers with the dominant (visible) allele inherited from ‘RS239’ could be mapped in the BC_1_ while all polymorphic markers could be used in the MDE population.

With 481 markers and a length of 1,943.2 cM the marker density of the MDE map can already be considered high. Nevertheless, there were four unassigned groups of two and three markers and A2 was present in two unjoined parts. In addition, only a short fragment of C4 was found. This indicates that some regions of the genome are not covered by markers in this map or that there are spots in the genome with exceptionally high recombination rates that would separate flanking regions in a genetic mapping.

About 48 % of the markers on the MDE map showed skewed segregations. High rates of skewed marker segregations are a common feature in DH populations. Analysing marker segregations in a cross between ‘Mansholt’s Hamburger Raps’ and ‘Samourai’, two winter rapeseed varieties, Uzunova et al. (1995) observed skewed segregations at 27.8 % of the marker loci. Working with three different populations Lombard and Delourme (2001) found rates ranging from 8.8 to 24.6 % and Zhao et al. (2005) observed skewed segregations at 35.2 % of marker loci in a cross between ‘Sollux’, a European winter rapeseed variety, and ‘Gaoyou’, a Chinese semi-winter variety. A similar high rate of 31.4 % was found by Radoev (2007) in a cross between ‘R53’, a resynthesized rapeseed, and ‘Express 617’ and Udall et al. (2005) observed a rate of 41 % (*P* ≤ 0.01), also in a cross between a resynthesized and a natural rapeseed genotype.

While the fraction of markers with skewed segregations on the MDE map was higher than any value reported in the literature, with only 12 % the rate of markers with skewed segregation on the BC_1_ map was rather low. Furthermore, in only five of the regions where markers with skewed segregations had clustered on the MDE map, skewed segregations were also observed on the BC_1_ map and in three of these regions the favoured allele was different. Since in the development of the BC_1_ population the F_1_ plant had been the pollinator, the marker segregations in this population are due to the same meiosis, the meiosis during the formation of microspores, as in the MDE population. The much higher rate of skewed segregations in the MDE population then can only be due to additional selective processes during embryogenesis, either the allelic segregation of genes that control the induction of a sporophytic development in isolated microspores or genes that affect the further development of the induced microspores. Both types of genes would contribute to the embryogenic potential as defined in this work.

The markers with skewed segregations clustered in 34 regions on the MDE map, suggesting a high number of genes affecting embryogenic potential segregating in the MDE population. Furthermore, some of these regions were rather long, which may indicate that they carry more than one gene causing skewed segregations. Since close to equal numbers of regions favoured ‘Express 617’ and ‘RS239’ marker alleles, both parents contributed similar numbers of alleles favourable for embryogenic potential. Accordingly, ISLs were selected with donor segments (partially) covering five regions with skewed segregations, two of which favoured ‘Express’ alleles and three favouring ‘RS239’ alleles.

To improve the genetic characterization of the selected ISLs, the lines were subjected to a high-throughput SNP analysis with the Illumina Infinium 60K chip for rapeseed. This analysis yielded more than 20,000 polymorphic markers of which 7,960 had been mapped in the SGDH14xE mapping population and could, therefore, been used for a genetic analysis of the ISLs. With two exceptions all donor segments previously found by the AFLP markers were detected again in this analysis. The exceptions were two segments, each represented by only one AFLP marker. With such one-marker segments it is always possible that they are due to scoring errors, not true donor segments, and would, accordingly, not been detected again in a different analysis. In addition to the already known donor segments, the SNP analysis detected 29 new segments. Most of these segments were small and were probably detected due to the much higher marker density of the SNP map—the 7960 markers represent more than 16 times the marker number on the DH map—but there was also a larger segment of 21.5 cM on A9 that also had not been detected in the AFLP analysis. This may indicate a somewhat incomplete genome coverage by the DH map.

The SNP analysis revealed donor segments in some of the ISLs carrying blocks of markers, some of which spanned several cM, where all marker assays had failed in the respective genotypes. A manual inspection showed very low assay signals, clearly indicating that these SNPs had actually been deleted from the affected lines. The deletions may indicate simple sequence losses. On the other hand, rapeseed is known for recombination between homoeologous segments of the A and C genomes, leading to an exchange of sequences between the genomes. Analysing the reference genome sequence of *B. napus* ‘Darmor-*bzh*’ Chalhoub et al. (2014) found homoeologous exchanges between the A and C genomes, where a sequence from one genome had been duplicated, replacing the corresponding sequence in the homoeologous region of the other genome. These exchanges were observed on different scales, ranging from 17 segmental exchanges covering hundreds of kilobases across 93 whole-gene conversions to a large number of small-scale exchanges apparent in the conversion of single nucleotide variants between homoeologous genes. These homoeologous exchanges, which result from the occasional pairing of homoeologous chromosomal segments during meiosis, are an ongoing process since new exchanges have been observed in segregating populations of rapeseed with low frequencies (Sharpe et al. 1995; Udall et al. 2005). There is also comprehensive evidence that these exchanges occur with much higher frequencies in resynthesized rapeseed genotypes and in crosses between such genotypes and natural rapeseed (Parkin et al. 1995; Song et al. 1995; Udall et al. 2005; Szadkowski et al. 2010; Chalhoub et al. 2014). Taking homoeologous exchanges into account the blocks of deleted SNPs may also be due to a replacement of the sequences carrying the SNPs by the corresponding sequences from homoeologous regions of the other genome.

Most of the segments showing the deletions probably started out as proper donor segments with the deletions or homoeologous exchanges occurring during the development of the ISLs since on linkage groups C2, C6 and C8 there were no failed scorings at the respective marker loci in the parents. On C2 there is actually a proper donor segment in ER165 from 0.0 to 10.4 cM that represents a subsegment of segments modified by deletions in ER137 and ER1437. Only on C1 large numbers of markers with failed scorings were observed in ‘RS239’. Here the segments with the deletions may represent a homoeologous exchange that pre-existed in the resynthesized parent although this is not a sure conclusion since the ‘RS239’ individual that was included in the SNP analysis was not the same individual that was crossed to develop the ISL population.

The segments on C1 and one segment on C2 had already been found in the AFLP analysis, but since AFLP markers are dominant markers this analysis could not show that parts of these segments are deleted. Being dominant may also explain why the other segments with extensive deletions were not detected by the AFLP markers, although some of these segments are rather large.

In addition to the continuous blocks of deleted markers there was a low background of dispersed irregular scorings, that is failed and heterozygous scorings. The simplest explanation for this background would be that marker assays sometimes fail or give erroneous results, as is commonly observed with many marker types. This explanation probably is true for the horizontal clustering, where a single marker shows irregular scorings across many lines, but cannot explain the vertical clustering observed in different genomic regions in just one or a few lines. The vertical clusterings of irregular scores may represent imprints of donor segments that were present in earlier generations during the development of the respective ISLs. Such donor segments may have undergone small-scale homoeologous recombinations, transferring donor alleles to homoeologous regions in the other genome. If in the final ISL the donor segment has been lost by genetic segregation but the homoeologous region has been retained, an SNP marker assay may detect both, the donor and the recurrent parent allele, reporting a seemingly heterozygous genotype. Likewise, failed marker scores may also be explained by homoeologous recombination, assuming that such an event has transferred a sequence from a homoeologous position to the marker position, thereby removing the sequence detected by the marker.

The recurrent parent of the ISL population, ‘Express 617’ showed a rather low embryogenic potential of 11.9 MDEs per 10^6^ cultivated microspores at the optimal stage. On the other hand, among the selected ISLs a broad range in the embryogenic potential was observed, ranging from 0.2 times to 40.4 times that of ‘Express 617’, representing a variation by a factor of 202. Ferrie and Keller (2007) have screened the embryogenic potential of 21 *B. napus* cultivars. They found a very broad range, covering 5 orders of magnitude. Their results are not directly comparable to our results since they expressed embryogenic potential as the number of embryos per 100 buds but recalculation of our data indicates that the range observed among the ISLs covers the lower tenth of the range observed by Ferrie and Keller. Nevertheless, eight of the 21 genotypes (38 %) analysed by Ferrie and Keller fall within this range, indicating that embryogenic potentials similar to the potentials of the selected ISLs are quite common among rapeseed varieties.

Although about equal numbers of regions with skewed segregations favouring ‘Express 617’ and ‘RS239’ alleles have been observed in the MDE population and seven ISLs had been selected because of segments covering regions with excess of ‘Express 617’ alleles, none of the 15 selected ISLs showed a significantly lower embryogenic potential than ‘Express 617’. The failure to detect significantly lower embryogenic potentials may be due to the smaller range of potentials below that of ‘Express 617’, just 0.9–0.2 times the potential of ‘Express 617’ compared to the range above ‘Express 617’ where embryogenic potentials up to 40.4 times higher than that of ‘Express 617’ were observed. In conjunction with the high standard deviations, in relation to the means, that reflect the broad distributions of values across repeated microspore cultivations observed for all genotypes, the smaller range may have prevented the detection of ISLs with significantly lower embryogenic potential than the recurrent parent.

The high standard deviations indicate that many repetitions are required to get a reliable estimate of the embryogenic potential of a genotype. This also illustrates the difficulty to determine the embryogenic potential of all lines of a reasonably sized mapping population and may explain the absence of publications, with the exception of the work by Zhang et al. (2003), where the hypothesis that skewed segregations in DH populations are due to selection at loci controlling embryogenic potential has been verified.

When analysing the embryogenic potential of the ISLs seven lines showed a significantly enhanced embryogenic potential compared to ‘Express 617’. By comparing donor segments between these seven significant lines and the remaining eight non-significant lines, 12 genomic regions were identified that were only covered by donor segments in the significant lines. Accordingly, these regions should carry the genetic factors giving the significant lines their enhanced embryogenic potential. The 15 ISLs have originally been selected because of donor segments that overlap five genomic regions that displayed strongly skewed marker segregations in the MDE population. In the two regions on A5 and A7, an excess of ‘Express 617’ alleles was observed. Donor alleles in these regions should decrease embryogenic potential. As expected, since no ISLs with significantly reduced embryogenic potential had been found, the donor segments covering these regions did not overlap with a region implicated in the control of embryogenic potential. Likewise, although selected because they cover a region on A2b with excess of ‘RS239’ alleles in the MDE population, the respective donor segments do not overlap with the region on A2 implicated in the control of embryogenic potential and the two ISL carrying these segments, ER792 and ER1284, did not show an enhanced embryogenic potential. On the other hand, the lower part from 2.4 to 28.0 cM of the donor segments on A2 in ER137 and ER228, which had been selected because they cover a region with skewed segregations on A2a of the MDE map, delineate the region on A2 implicated in the control of embryogenic potential. Similarly, the segments on A4 in lines ER36 and ER165 define the respective region on A4. These results support the hypothesis that the skewed segregations in the MDE population on A2a and A4 are caused by the segregation of alleles at loci controlling embryogenic potential. The results do not prove this hypothesis because all four lines carry additional segments covering or overlapping the regions implicated in the control of embryogenic potential, although the two additional segments in ER137 are rather small making it more likely that the effective factor resides on the larger segment on A2.

In addition to the four lines with segments covering the regions with skewed segregations on A2a and A4 three additional lines, ER85, ER1129, and ER1321, showed a significantly enhanced embryogenic potential. Although these lines had been selected for segments covering regions favouring ‘Express 617’ alleles in the MDE population, they must carry alleles that strongly increase embryogenic potential, reinforcing the notion that many loci controlling embryogenic potential are segregating in the cross of ‘Express 617’ and ‘RS239’.

Among the genomic regions implicated in the control of embryogenic potential are two, on C1 and C8, which are defined by donor segments containing blocks of deleted SNPs. The segment on C1 from 0 to 20.4 cM is actually present in three lines and a subsegment from 0 to 14.3 cM in a fourth line. Among the donor segments in ER85, the ISL with the highest embryogenic potential of all 15 lines, only these two donor segments are not shared with non-significant lines. This result indicates that, in addition to genetic diversity already present in the parents, new diversity created during the development of the ISLs also contributed to the broad range of embryogenic potentials seen in the 15 ISLs, since at least the deletions or homoeologous exchanges on C8 were not pre-existing in ‘RS239’ and possibly also not on C1.

In the work presented it was possible to identify 12 genomic regions, which may carry genetic factors affecting embryogenic potential in isolated microspores of rapeseed. It was not possible to assign such a factor unambiguously to a specific region since, with the exception of ER1129, the ISL with significantly enhanced embryogenic potential carry donor segments that overlap with at least two of the 12 genomic regions. This may not be due to chance because several lines of evidence indicate that the cross ‘Express 617’ × ‘RS239’ segregates for a large number of factors affecting embryogenic potential. Taking this into account, it may be that in many of the significant ISLs more than one genetic factor contributes to the enhanced embryogenic potential. In ER1129, on the other hand, no segment overlapping one of the regions implicated in the control of embryogenic potential was observed, but this line carries a long cluster of irregular scores on A4 that also covers the respective region on that linkage group.

Although no single genomic region surely involved in controlling embryogenic potential could be identified, seven ISLs with significantly enhanced embryogenic potentials covering a rather large range in this trait were found. These lines differ genetically from ‘Express 617’ only in a limited way. Just 1.5–5.29 % of the genome is covered in donor segments in these lines with the irregular scores indicating additional differences in up to 2 %. This close genetic similarity to ‘Express 617’ makes these lines an ideal material for further genetic analyses into the control of embryogenic potential. Starting with these lines it should be possible to fine map the controlling factors by backcrossing the lines again with ‘Express 617’ and developing lines with fewer donor segments and with the segments implicated in the control of embryogenic potential subdivided. It should also be quite illuminating to use the lines to study how transcription, protein and metabolic patterns differ between the lines and ‘Express 617’ or change with increasing embryogenic potential.

### **Author contribution statement**

KZK was involved in the development of the MDE and BC1 populations and together with AK did the AFLP analyses in these populations. WE carried out the map constructions while AK analysed the skewed segregations and selected the ISLs. KZK, AK, ACH determined the embryogenic potential of the ISLs and ACH and WE carried out the statistical analysis of the results. WE was responsible for the analysis and evaluation of the results from the high-throughput SNP analysis. WE also designed the project and provided guidance and discussions throughout the project.

## Electronic supplementary material

Below is the link to the electronic supplementary material.

Supplementary material 1 (XLSX 1136 kb)

Supplementary material 2 (PDF 1995 kb)
