# Supplementary material for: Identification and genetic characterization by high-throughput SNP analysis of intervarietal substitution lines of rapeseed (Brassica napus L.) with enhanced embryogenic potential
Source: Theor Appl Genet. 2015 Jan 28;128(4):587–603. doi: 10.1007/s00122-015-2455-7 (PMC4361729; doi:10.1007/s00122-015-2455-7)
Supplement: Supplementary file 2 — Supplementary material 2 (PDF 1995 kb) [file 122_2015_2455_MOESM2_ESM.pdf]

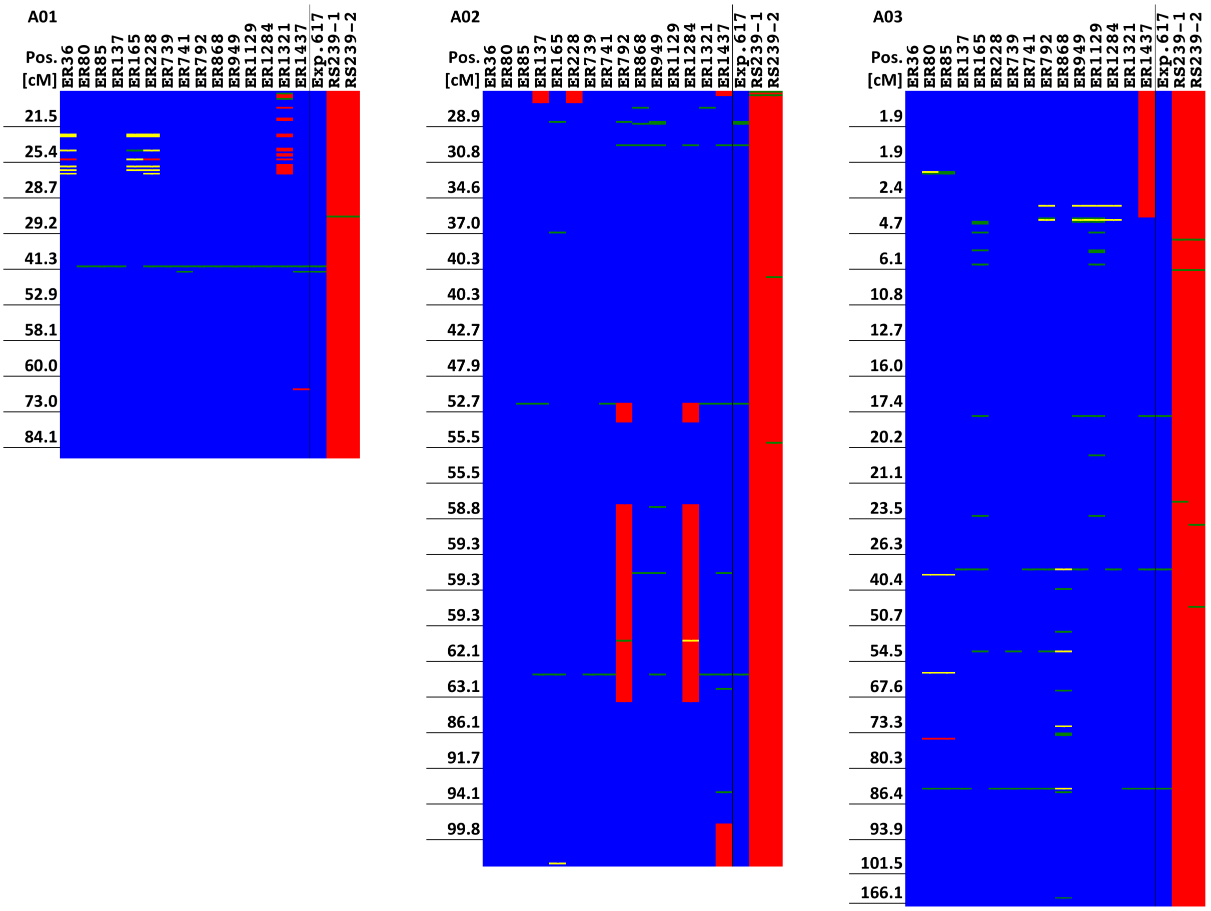

Figure S1: Overview of donor segments and marker scorings in the selected ISLs and the parents on the linkage groups of the SGDH14xE map

Blue and red color indicate alleles of the recurrent and donor parent, respectively, green and yellow color failed and heterozygous scorings. Exp.617 is the recurrent parent 'Express 617', RS239-1 and RS239-2 are two replicates of the same DNA sample of 'RS239' in the SNP analysis.

Marker positions are given for every 20th marker.

Reference: Theoretical and Applied Genetics, Wolfgang Ecke, Anthimos Kampouridis, Katharina Ziese-Kubon, Ann-Catrin Hirsch, Identification and genetic characterization by high-throughput SNP analysis of intervarietal substitution lines of rapeseed (Brassica napus L.) with enhanced embryogenic potential, Georg-August-Universität Göttingen, Department of Crop Sciences, Von-Siebold-Str. 8, Göttingen, 37075, Germany, Corresponding author: Wolfgang Ecke (wecke@gwdg.de)

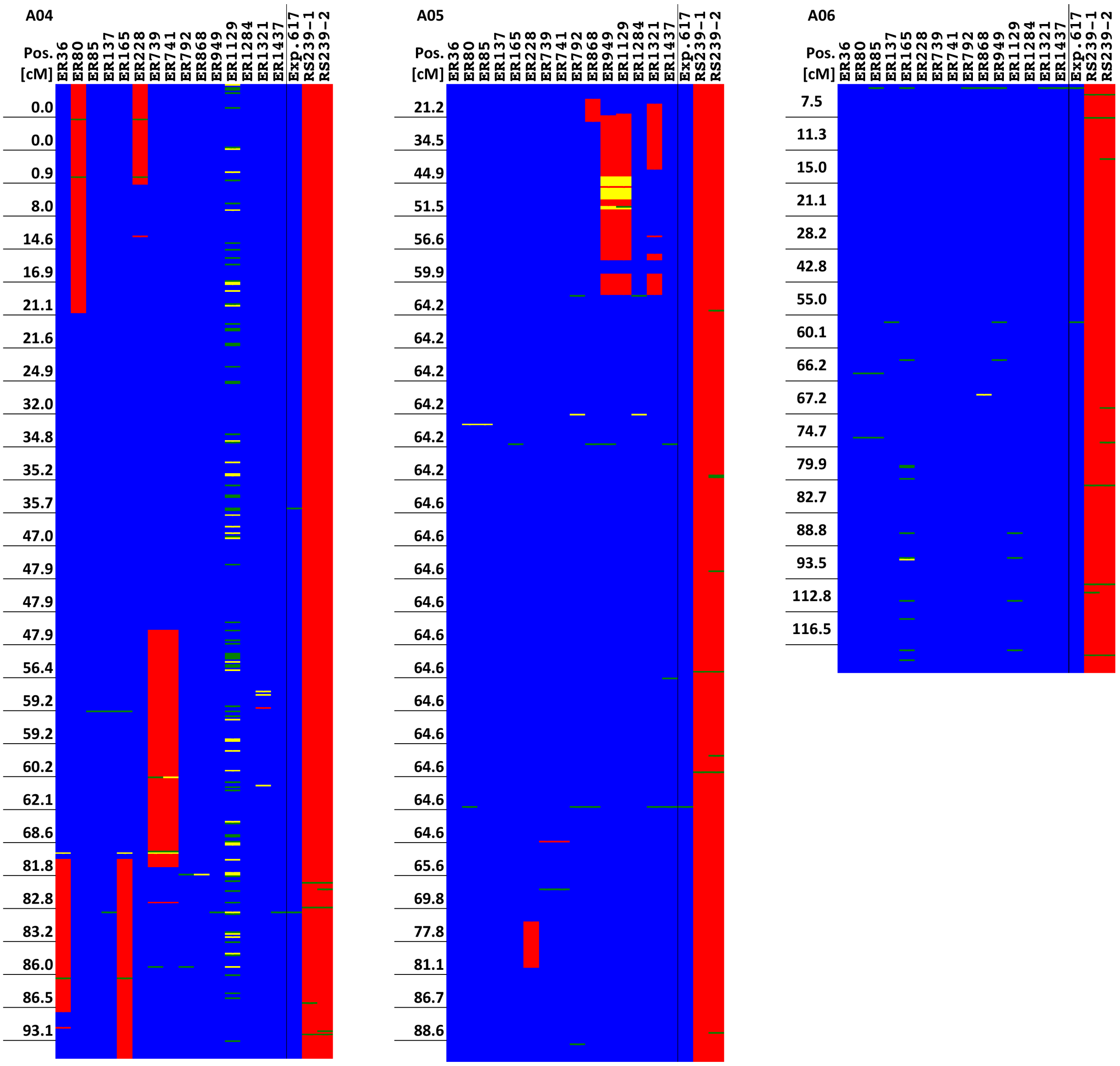
A04
Pos. [cM]
ER36 ER80 ER85 ER137 ER165 ER228 ER739 ER741 ER792 ER868 ER949 ER1129 ER1284 ER1321 ER1437 Exp.617 RS239-1 RS239-2
0.0 0.0 0.9 8.0 14.6 16.9 21.1 21.6 24.9 32.0 34.8 35.2 35.7 47.0 47.9 47.9 47.9 56.4 59.2 59.2 60.2 62.1 68.6 81.8 82.8 83.2 86.0 86.5 93.1
A05
Pos. [cM]
ER36 ER80 ER85 ER137 ER165 ER228 ER739 ER741 ER792 ER868 ER949 ER1129 ER1284 ER1321 ER1437 Exp.617 RS239-1 RS239-2
21.2 34.5 44.9 51.5 56.6 59.9 64.2 64.2 64.2 64.2 64.2 64.2 64.6 64.6 64.6 64.6 64.6 64.6 64.6 64.6 64.6 64.6 64.6 65.6 69.8 77.8 81.1 86.7 88.6
A06
Pos. [cM]
ER36 ER80 ER85 ER137 ER165 ER228 ER739 ER741 ER792 ER868 ER949 ER1129 ER1284 ER1321 ER1437 Exp.617 RS239-1 RS239-2
7.5 11.3 15.0 21.1 28.2 42.8 55.0 60.1 66.2 67.2 74.7 79.9 82.7 88.8 93.5 112.8 116.5

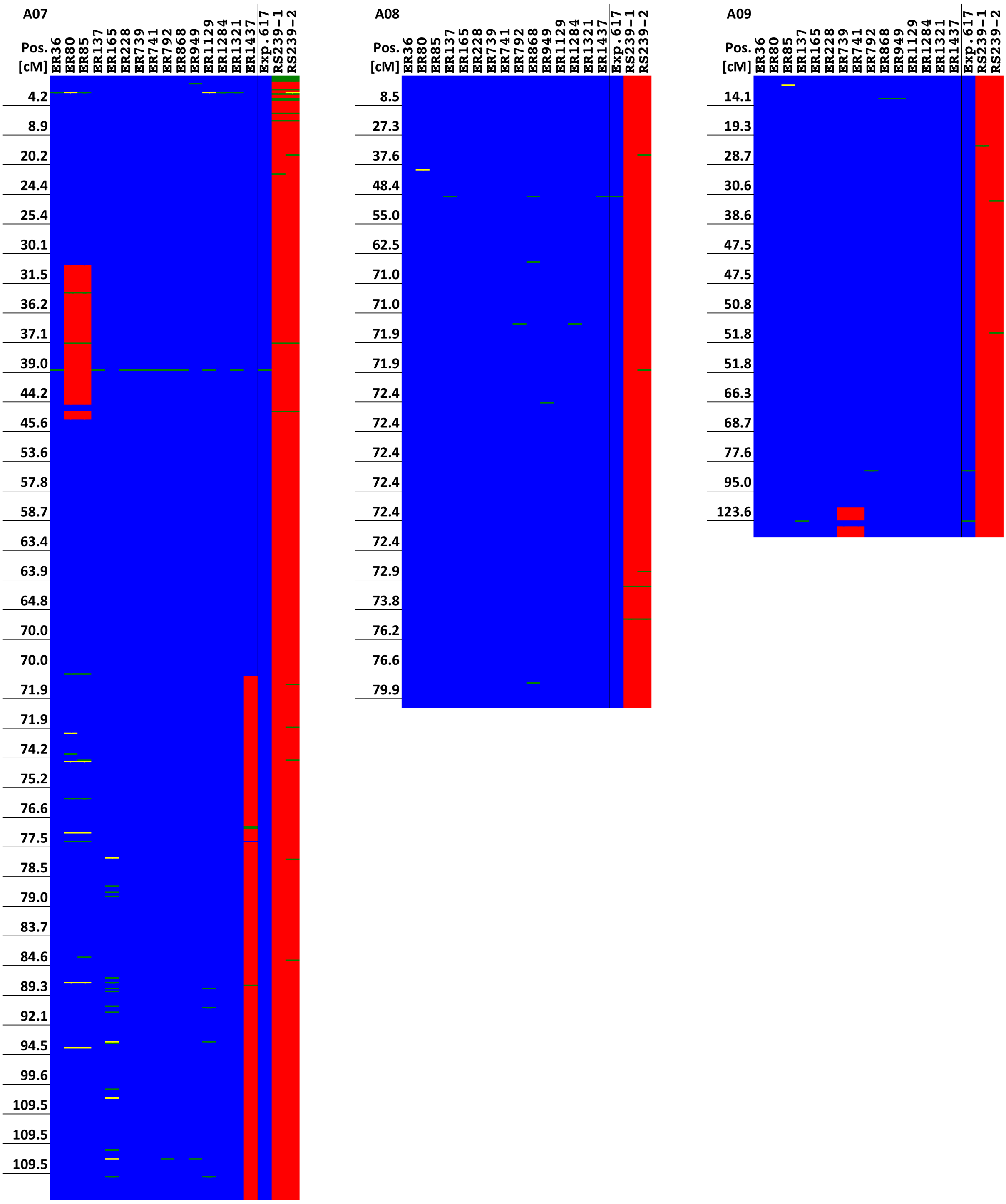
A07
Pos. [cM]
ER36
ER80
ER85
ER137
ER165
ER228
ER739
ER741
ER792
ER868
ER949
ER1129
ER1284
ER1321
ER1437
Exp.617
RS239-1
RS239-2
4.2
8.9
20.2
24.4
25.4
30.1
31.5
36.2
37.1
39.0
44.2
45.6
53.6
57.8
58.7
63.4
63.9
64.8
70.0
70.0
71.9
71.9
74.2
75.2
76.6
77.5
78.5
79.0
83.7
84.6
89.3
92.1
94.5
99.6
109.5
109.5
109.5
A08
Pos. [cM]
ER36
ER80
ER85
ER137
ER165
ER228
ER739
ER741
ER792
ER868
ER949
ER1129
ER1284
ER1321
ER1437
Exp.617
RS239-1
RS239-2
8.5
27.3
37.6
48.4
55.0
62.5
71.0
71.0
71.9
71.9
72.4
72.4
72.4
72.4
72.4
72.4
72.9
73.8
76.2
76.6
79.9
A09
Pos. [cM]
ER36
ER80
ER85
ER137
ER165
ER228
ER739
ER741
ER792
ER868
ER949
ER1129
ER1284
ER1321
ER1437
Exp.617
RS239-1
RS239-2
14.1
19.3
28.7
30.6
38.6
47.5
47.5
50.8
51.8
51.8
66.3
68.7
77.6
95.0
123.6

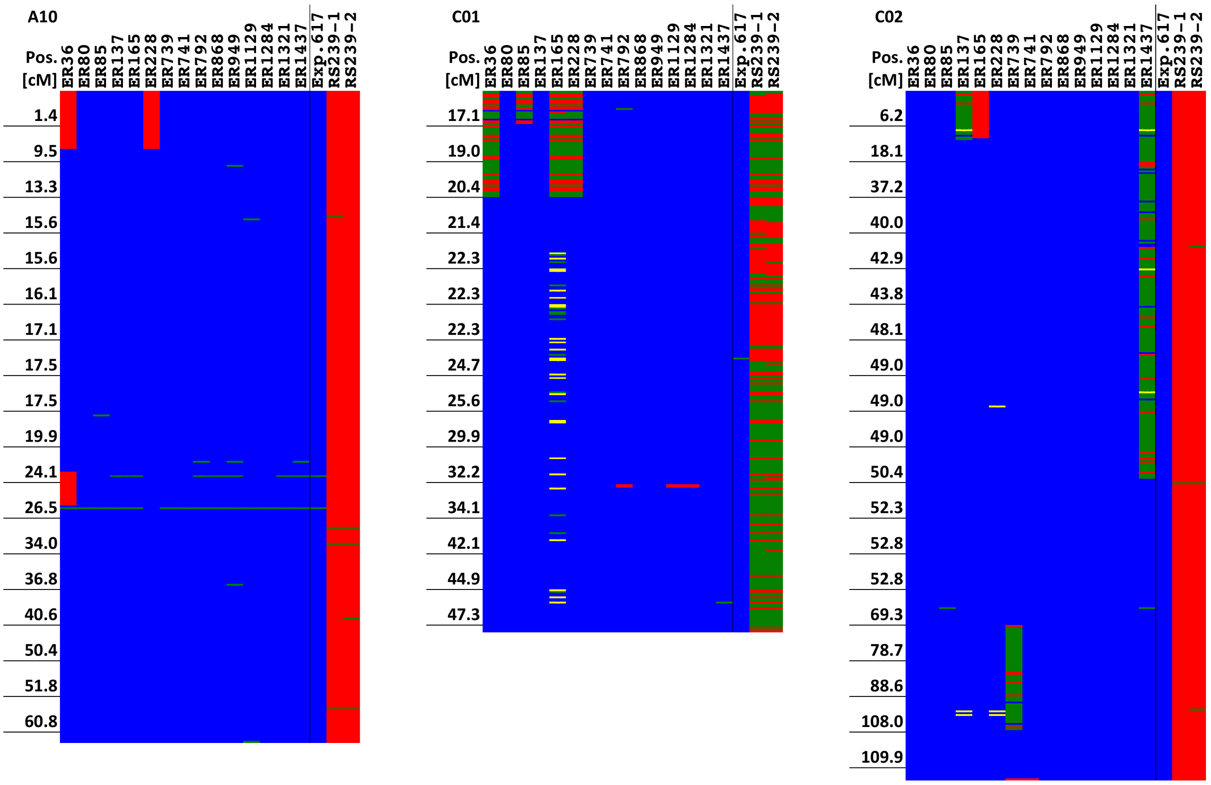

A10
Pos. [cM]
ER36 ER80 ER85 ER137 ER165 ER228 ER739 ER741 ER792 ER868 ER949 ER1129 ER1284 ER1321 ER1437 Exp.617 RS239-1 RS239-2
1.4 9.5 13.3 15.6 15.6 16.1 17.1 17.5 17.5 19.9 24.1 26.5 34.0 36.8 40.6 50.4 51.8 60.8
C01
Pos. [cM]
ER36 ER80 ER85 ER137 ER165 ER228 ER739 ER741 ER792 ER868 ER949 ER1129 ER1284 ER1321 ER1437 Exp.617 RS239-1 RS239-2
17.1 19.0 20.4 21.4 22.3 22.3 22.3 24.7 25.6 29.9 32.2 34.1 42.1 44.9 47.3
C02
Pos. [cM]
ER36 ER80 ER85 ER137 ER165 ER228 ER739 ER741 ER792 ER868 ER949 ER1129 ER1284 ER1321 ER1437 Exp.617 RS239-1 RS239-2
6.2 18.1 37.2 40.0 42.9 43.8 48.1 49.0 49.0 49.0 50.4 52.3 52.8 52.8 69.3 78.7 88.6 108.0 109.9

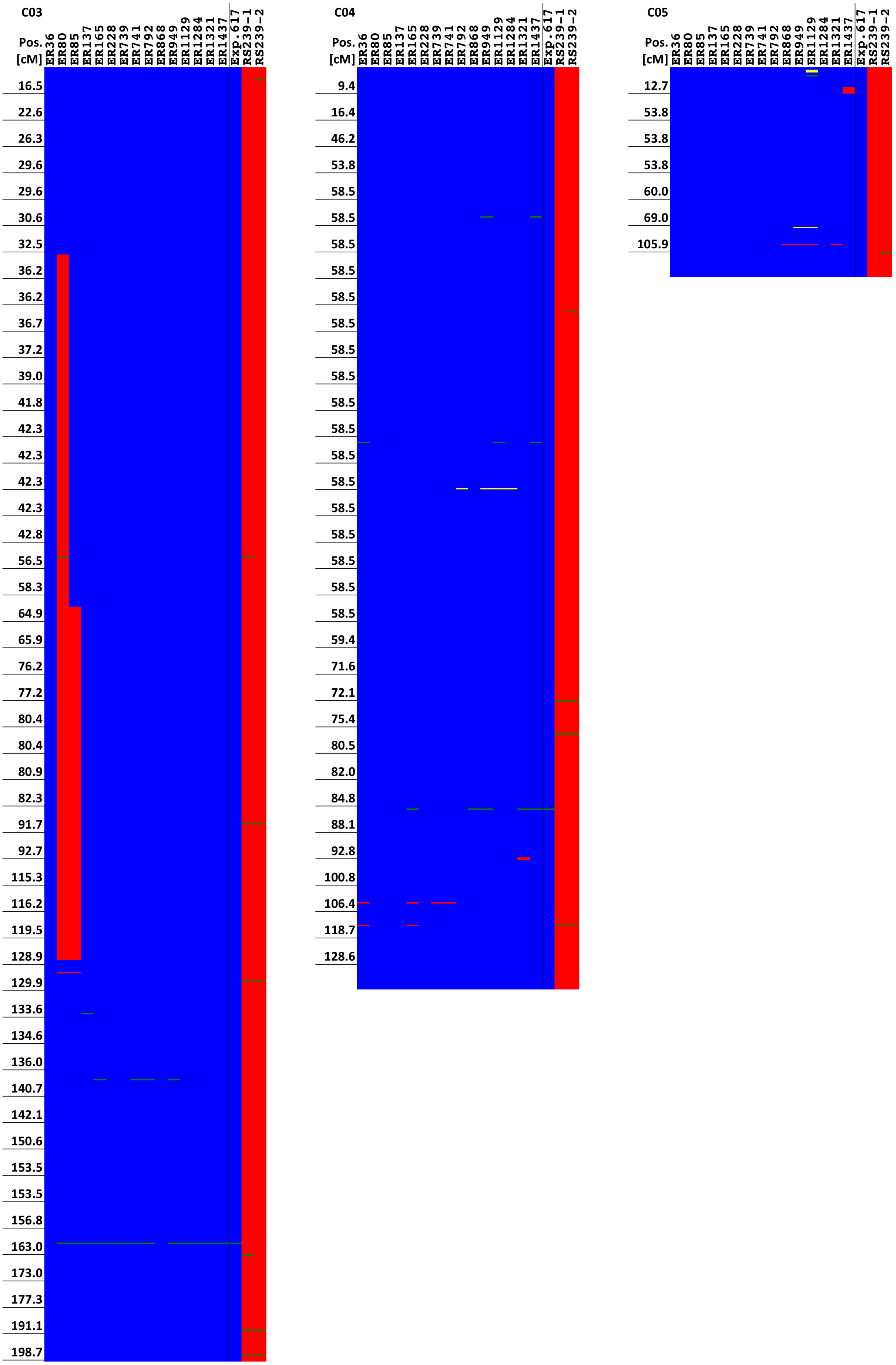
C03
Pos. [cM]
ER36
ER80
ER85
ER137
ER165
ER228
ER739
ER741
ER792
ER868
ER949
ER1129
ER1284
ER1321
ER1437
Exp.617
RS239-1
RS239-2
16.5
22.6
26.3
29.6
29.6
30.6
32.5
36.2
36.2
36.7
37.2
39.0
41.8
42.3
42.3
42.3
42.3
42.8
56.5
58.3
64.9
65.9
76.2
77.2
80.4
80.4
80.9
82.3
91.7
92.7
115.3
116.2
119.5
128.9
129.9
133.6
134.6
136.0
140.7
142.1
150.6
153.5
153.5
156.8
163.0
173.0
177.3
191.1
198.7
C04
Pos. [cM]
ER36
ER80
ER85
ER137
ER165
ER228
ER739
ER741
ER792
ER868
ER949
ER1129
ER1284
ER1321
ER1437
Exp.617
RS239-1
RS239-2
9.4
16.4
46.2
53.8
58.5
58.5
58.5
58.5
58.5
58.5
58.5
58.5
58.5
58.5
58.5
58.5
58.5
58.5
58.5
58.5
58.5
59.4
71.6
72.1
75.4
80.5
82.0
84.8
88.1
92.8
100.8
106.4
118.7
128.6
C05
Pos. [cM]
ER36
ER80
ER85
ER137
ER165
ER228
ER739
ER741
ER792
ER868
ER949
ER1129
ER1284
ER1321
ER1437
Exp.617
RS239-1
RS239-2
12.7
53.8
53.8
53.8
60.0
69.0
105.9

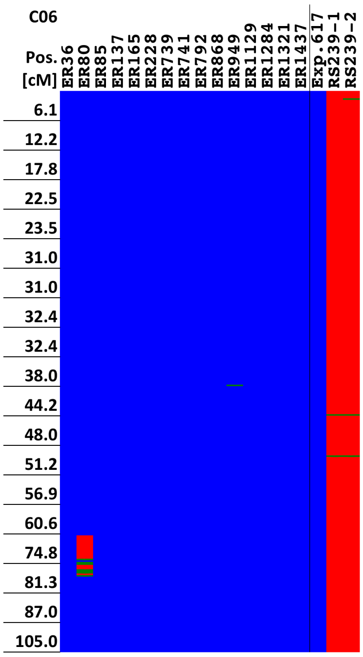
C06
Pos. [cM]
ER36
ER80
ER85
ER137
ER165
ER228
ER739
ER741
ER792
ER868
ER949
ER1129
ER1284
ER1321
ER1437
Exp.617
RS239-1
RS239-2
6.1
12.2
17.8
22.5
23.5
31.0
31.0
32.4
32.4
38.0
44.2
48.0
51.2
56.9
60.6
74.8
81.3
87.0
105.0

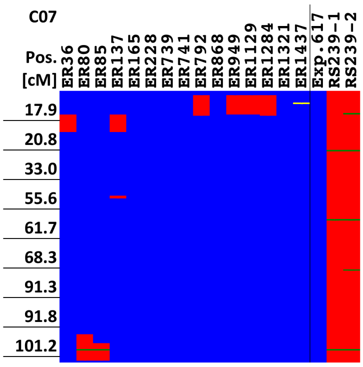
C07
Pos. [cM]
ER36
ER80
ER85
ER137
ER165
ER228
ER739
ER741
ER792
ER868
ER949
ER1129
ER1284
ER1321
ER1437
Exp.617
RS239-1
RS239-2
17.9
20.8
33.0
55.6
61.7
68.3
91.3
91.8
101.2

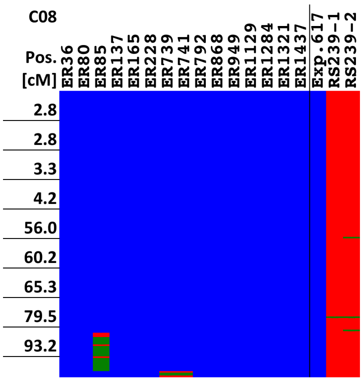
C08
Pos. [cM]
ER36
ER80
ER85
ER137
ER165
ER228
ER739
ER741
ER792
ER868
ER949
ER1129
ER1284
ER1321
ER1437
Exp.617
RS239-1
RS239-2
2.8
2.8
3.3
4.2
56.0
60.2
65.3
79.5
93.2

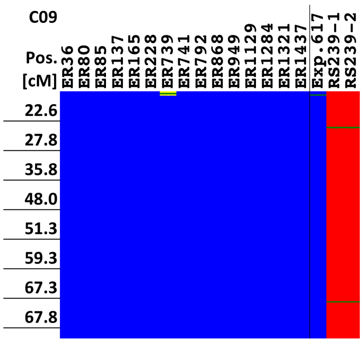
C09
Pos. [cM]
ER36
ER80
ER85
ER137
ER165
ER228
ER739
ER741
ER792
ER868
ER949
ER1129
ER1284
ER1321
ER1437
Exp.617
RS239-1
RS239-2
22.6
27.8
35.8
48.0
51.3
59.3
67.3
67.8
